# Fecal transplantation alleviates acute liver injury in mice through regulating Treg/Th17 cytokines balance

**DOI:** 10.1038/s41598-021-81263-y

**Published:** 2021-01-15

**Authors:** Yongmei Liu, Linda Fan, Zhuo Cheng, Lei Yu, Shuo Cong, Yaxin Hu, Lili Zhu, Baofang Zhang, Yiju Cheng, Peiling Zhao, Xueke Zhao, Mingliang Cheng

**Affiliations:** 1 Department of Medical Examination, Guizhou Medical University, Guiyang, Guizhou China; 2 Clinical Laboratory Center, The Affiliated Hospital of Guizhou Medical University, Guiyang, Guizhou China; 3 Department of Infectious Diseases, The Affiliated Hospital of Guizhou Medical University, No. 28 Guiyang Street, Guiyang, 550002 Guizhou China; 4 Department of Clinical Medicine, Peking University Health Science Center School of Foundational Education, Peking University, Beijing, China; 5 Guizhou Maternal and Child Health Care Center, Guiyang, Guizhou China; 6 Deparment of Blood Transfusion, The Affiliated Tumor Hospital, Guizhou Medical University, Guiyang, Guizhou China; 7 Prenatal Diagnosis Center, The Affiliated Hospital of Guizhou Medical University, Guiyang, Guizhou China; 8 Department of Blood Transfusion, The Affiliated Hospital of Guizhou Medical University, Guiyang, Guizhou China; 9 Department of Respiratory, The Affiliated Hospital of Guizhou Medical University, Guiyang, Guizhou China

**Keywords:** Microbiology, Diseases

## Abstract

Changes in intestinal microecology during acute liver failure (ALF) directly affect the occurrence and development of the disease. The study aimed to investigate the relationship between the intestinal microbiota and the key immune cells. Fecal microbiota transplantation (FMT) was used to determine whether ALF can balance Th17/Treg cytokines. The relationship between gut microbiota and clinical indicators was analyzed. BALB/c mice were treated with d-galactosamine (d-GalN) to induce a murine ALF model. FMT to d-GalN mice was conducted to test for liver function indicators. Results showed that the proportions of *Lachnospiraceae*, *Prevotella*, *S24-7*, *Odoribacter* and *Rikenellaceae* in d-GalN mice with intestinal microbiota disorder were restored after FMT. Further, CIA analysis showed that bacteria had a covariant relationship with clinical indicators. Microbiota could account for changes in 49.9% of the overall clinical indicators. Adonis analysis showed that *Ruminococcus*, and *Enterococcus* have a greater impact on clinical indicators. FMT down-regulated the expression of IL-17A, TNF-α, and TGF-β, while up-regulated IL-10 and IL-22. Transplantation of feces from *Saccharomyces boulardii* donor mice improved GalN-induced liver damage. These findings indicate that FMT attenuates d-GalN-induced liver damage in mice, and a clinical trial is required to validate the relevance of our findings in humans, and to test whether this therapeutic approach is effective for patients with ALF.

## Introduction

Acute liver failure (ALF) is a rare but life-threatening clinical syndrome characterized by the rapid death of large numbers of hepatocytes because of cell necrosis or apoptosis^1^. This loss of cells ultimately leads to functional impairment of the liver. The most common factors that contribute to the development of ALF are hepatitis, medication overdose, idiosyncratic drug reactions, and metabolic disorders^2^. Liver transplant remains the most effective solution for treating ALF. However, the scope of this treatment is limited by extremely high cost and shortage of liver donors, and hence the development of alternative therapies is urgently needed^3^.

The intestinal microbiota is inseparable from the body, whose activities are linked to numerous host functions^4–7^. These bacteria contribute to the microenvironment of the intestine via their metabolic activities. Accumulated evidence has demonstrated the important effects of gut microbes on disease, and has provided the rationale behind the hypothesis that managing microbiota may help treat liver diseases^8–10^. Previous studies reported that the gut microbiota play an important role in the pathogenesis of liver diseases. In a BALB/c mouse model, studies have indicated that metabolites produced by gut microbiota are responsible for the diurnal variation of acetaminophen-induced hepatotoxicity^11^. Studies have also shown that Rhubarb extract prevent alcohol-induced hepatic inflammation by modifying the gut microbiota^12^. In addition, T helper 17 (Th17) and regulatory T cells (Treg) are key immune cell subsets that link gut microbiota and intestinal immune function. Recent evidence suggests that changes in Th17/Treg balance plays a major role in the development and progression of inflammatory diseases^13^. Thus, the effects of fecal microbiota transplantation (FMT) on Th17/Treg and gut microbiota need to be investigated.

In recent years, FMT has been widely studied as an alternative treatment. Ferrere et al. showed that fecal transplant from alcohol-resistant donor mice restore gut homeostasis and prevent alcoholic liver disease^14^. Wang et al. reported that FMT prevents hepatic encephalopathy in a rat model^15^. Using humanized mice, Duan et al. reported that bacteriophages can specifically target cytolytic *E. faecalis*, which decrease cytolysin in the liver and abolish ethanol-induced liver disease^16^. Furthermore, a clinical trial showed that FMT from a rationally selected donor reduced hospitalizations, improved cognition, and dysbiosis in cirrhosis with recurrent hepatic encephalopathy^17^. However, there is limited evidence about the effects of FMT on ALF. In our previous study, we demonstrated that *Saccharomyces boulardii* administration can change the gut microbiota and attenuate drug-induced liver injury in a rodent model^18^. To further investigate whether the regulation of intestinal microbiota by *Saccharomyces boulardii* alleviate liver failure, we performed this study.We hypothesized that an association exists between gut microbiome and the outcome of d-galactosamine (d-GalN)-induced ALF. To test this hypothesis, we used fecal bacteria transplantation to treat mice with ALF, and analyzed liver function, pathology, and serum cytokines. 16S rDNA sequencing was performed to detect intestinal microbial diversity, and qRT-PCR, western blotting, and immunohistochemistry were performed to verify Th17/Treg cytokines. Overall, we provide an experimental basis for evaluating the potential clinical application of FMT in ALF patients.

## Results

### Fecal transplantation alters serum AST, ALT, and TBIL levels

**Figure 1:**
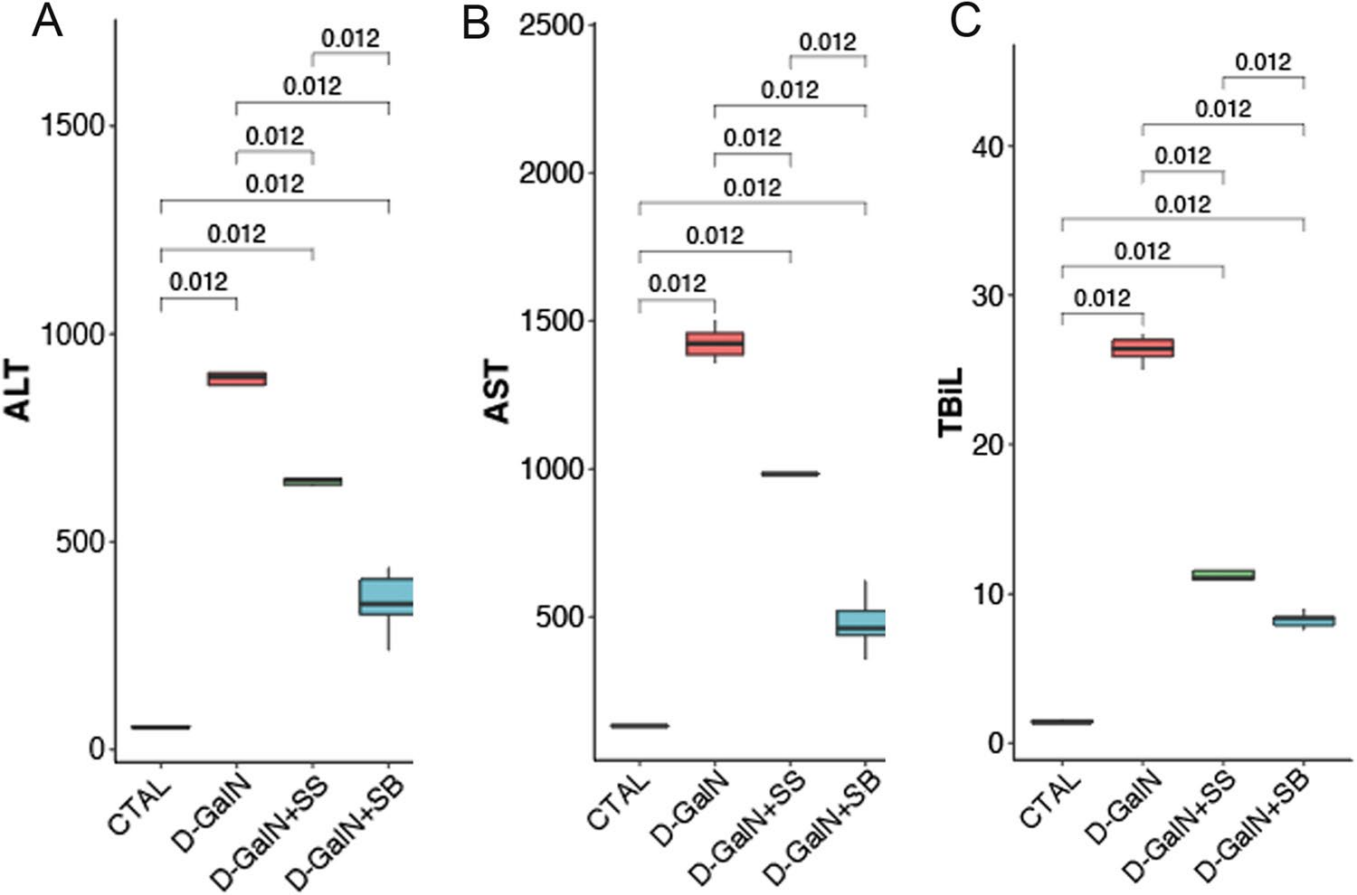
Effects of fecal microbiota transplantation (FMT) on (**A**) ALT, (**B**) AST, and (**C**) TBIL activities.

Four groups of mice were studied, including a CTRL group, a d-GalN group with d-GalN-induced liver injury, a d-GalN + SS group and a d-GalN + SB group with FMT treatment at d-GalN challenge. Plasma levels of ALT, AST, and TBIL were measured as an indicator of d-GalN-induced liver injury (Fig. 1). Compared with the CTRL group, there was a significant elevation in the levels of ALT, AST, and TBIL in the d-GalN group (*p* < 0.05 for ALT, AST), indicating massive abnormality in liver function. However, ALT, AST, and TBIL levels in the d-GalN + SS and d-GalN + SB group were significantly lower than those in the d-GalN group (*p* < 0.05); the levels decreased significantly in d-GalN + SB group compared with d-GalN + SS group (*p* < 0.05), suggesting marked attenuation in liver injury after FMT. The d-GalN + SB group was superior to the d-GalN + SS group (*p* < 0.05).

### Fecal transplantation alters serum IL-10, IL-17A, TNF-α, and TGF-β levels

IL-10, IL-17A, TNF-α, and TGF-β levels in the serum of mice are shown in Table 1. Compared with serum IL-10 and TNF-α levels in d-GalN group, those in the d-GalN + SS and the d-GalN + SB groups were significantly increased (*p* < 0.05), while IL-17A and TGF-β levels were significantly reduced (*p* < 0.05). Compared with serum IL-17A and TGF-β levels in d-GalN + SS group, those in d-GalN + SB group were significantly decreased (*p* < 0.05).

**Table 1:** The effects of fecal microbiota transplantation on serum IL-10, IL-17A, TNF-α, and TGF-β levels in mice.

Types	IL-10 (pg/ml)	IL-17A (pg/ml)	TNF-α(ng/ml)	TGF-β (ng/ml)
CTRL	25.34 ± 4.48	11.98 ± 2.92	395.39 ± 26.57	26.23 ± 6.51
d-GalN	9.59 ± 3.15*	30.58 ± 6.09*	770.26 ± 98.67*	229.79 ± 45.03*
d-GalN + SS	14.10 ± 2.68*#	19.73 ± 3.04*#	513.26 ± 31.31*#	155.34 ± 29.62*#
d-GalN + SB	15.95 ± 2.07*#	15.13 ± 2.75*#△	495.92 ± 40.71*#	108.12 ± 7.87*#△

*P < 0.05 compared with control group.

^#^*P* < 0.05 compared with d-GalN group.

^△^*P* < 0.05 compared with d-GalN + SS group.

### Histopathological analysis of liver tissues after fecal transplantation

**Figure 2:**
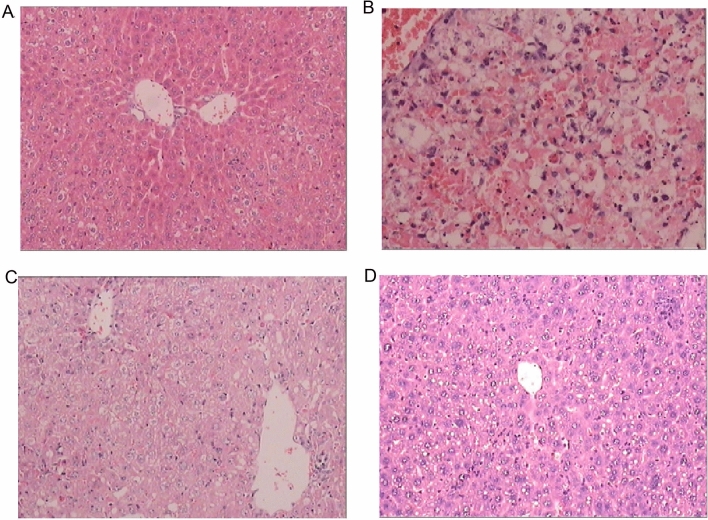
Microscopic examination of liver sections after FMT. (**A**) Histology of control group with abnormal liver architecture. (**B**) Histologically, d-GalN group showed obvious hemorrhage and inflammation, necrosis of hepatocytes, and severe dissolution of hepatocyte structure. (**C**) d-GalN-induced damage was partially restored in d-GalN + SS group. (**D**) Hepatic injury was markedly attenuated in d-GalN + SB group.

Histology of the mice liver sections exhibited a normal lobular liver architecture and integrated cell structure in CTRL group (Fig. 2A). Challenge with d-GalN resulted in acute hepatic injury accompanied by prominent hemorrhage and inflammation, necrosis of hepatocytes, and serious dissolution of the hepatocyte architecture (Fig. 2B). Such liver alterations were alleviated in the d-GalN + SS group and d-GalN + SB group (Fig. 2C,D). Such differences indicated that FMT from proper donors indeed alleviated GalN-induced liver injury and restored hepatocyte structure.

### Changes in gut microbiota after fecal transplantation

**Figure 3:**
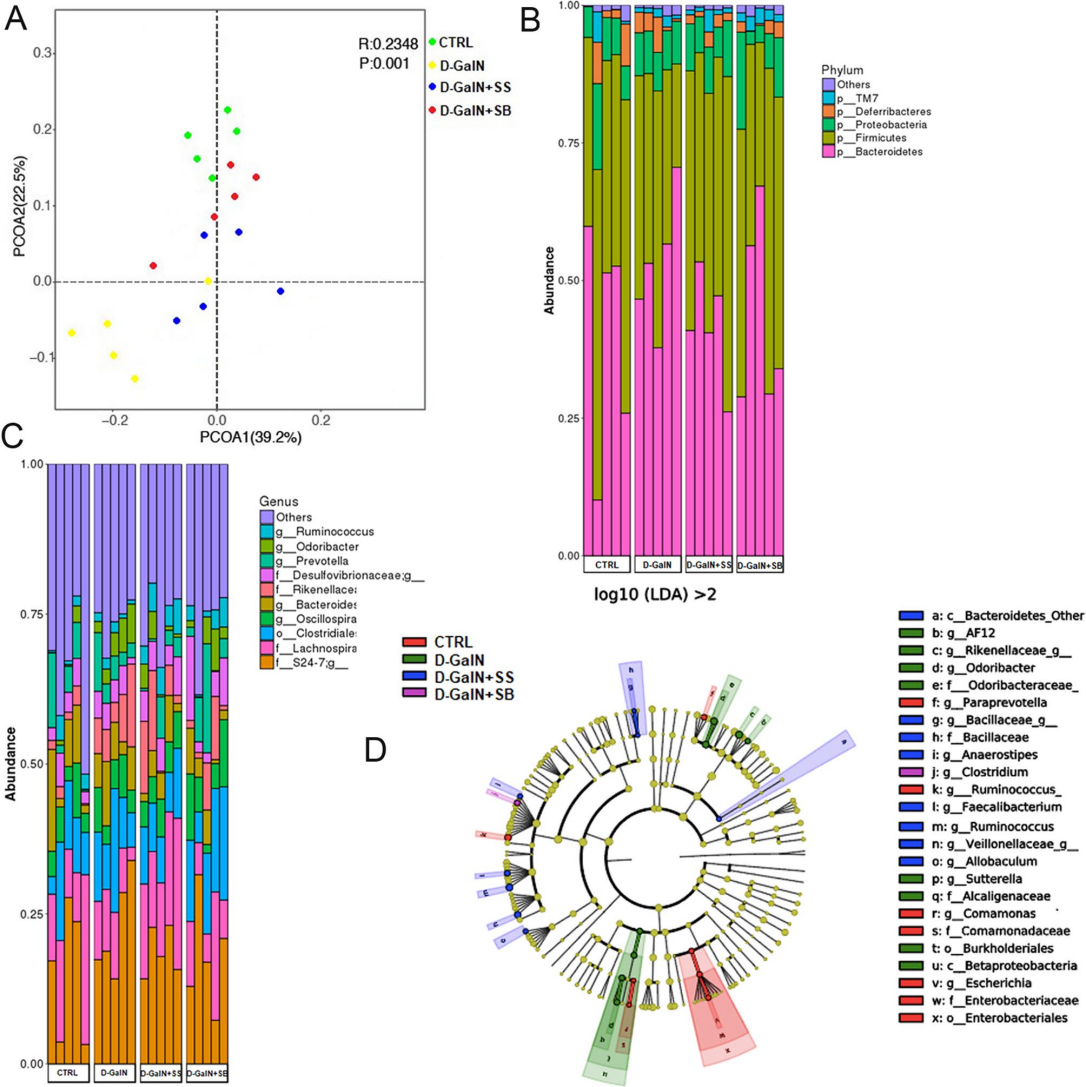
(**A**) Principal component analysis. (**B**) Gut microbiota at the pylums level. (**C**) Gut microbiota at the family level. (**D**) Results of LEfSe analysis.

In order to further study the underlying mechanisms for the protective effects of FMT, we performed 16S rDNA sequencing and examined the temporal changes to the composition of gut microbiota. The metagenomic DNA was quantified and mapped into operational taxonomic units (OTUs) for analysis. A principal coordinate analysis (PCoA) of Bray–Curtis dissimilarities (Fig. 3A) showed clear separation between samples collected after FMT in the d-GalN + SS and d-GalN + SB groups, indicating a change in gut microbiota.

At the phylum level, we found that the d-GalN + SB group was associated with a significant decrease in the relative abundance of *Bacteroidetes* and a significant increase in *Firmicutes* and *Proteobacteria* compared to the d-GalN group (*p* < 0.05, Fig. 3B). These results suggested that FMT from the donor mice gavaged with *Saccharomyces boulardii* changed the gut microbial community by altering the proportion of three major phyla. At the family level, we also observed several important modifications of the gut microbial composition. Among the major taxonomic families identified, *Lachnospiraceae* and *Prevotella* were increased following FMT treatment. Conversely, *S24-7*, *Odoribacter*, and *Rikenellaceae* were significantly decreased (*p* < 0.05, Fig. 3C).

LEfSe analysis of d-GalN group and d-GalN + SB group revealed that the cladogram and reflecting relative bacterial abundance in the d-GalN + SB group (Fig. 3D) exhibited that *Staphylococcus* and *Parasutterella* were overrepresented genera after FMT. Lefse analyses for d-GalN + SS versus d-GalN + SB and d-GalN versus d-GalN + SS were shown in Supplementary Fig. S1. In contrast, a decrease of *Anaeroplasma* and *Ruminococcus* genera occurred in response to FMT. Such temporal changes may explain the microbial mechanisms underlying the protective effects of FMT.

### Relationship between microbiota and clinical indicators after fecal transplantation

**Figure 4:**
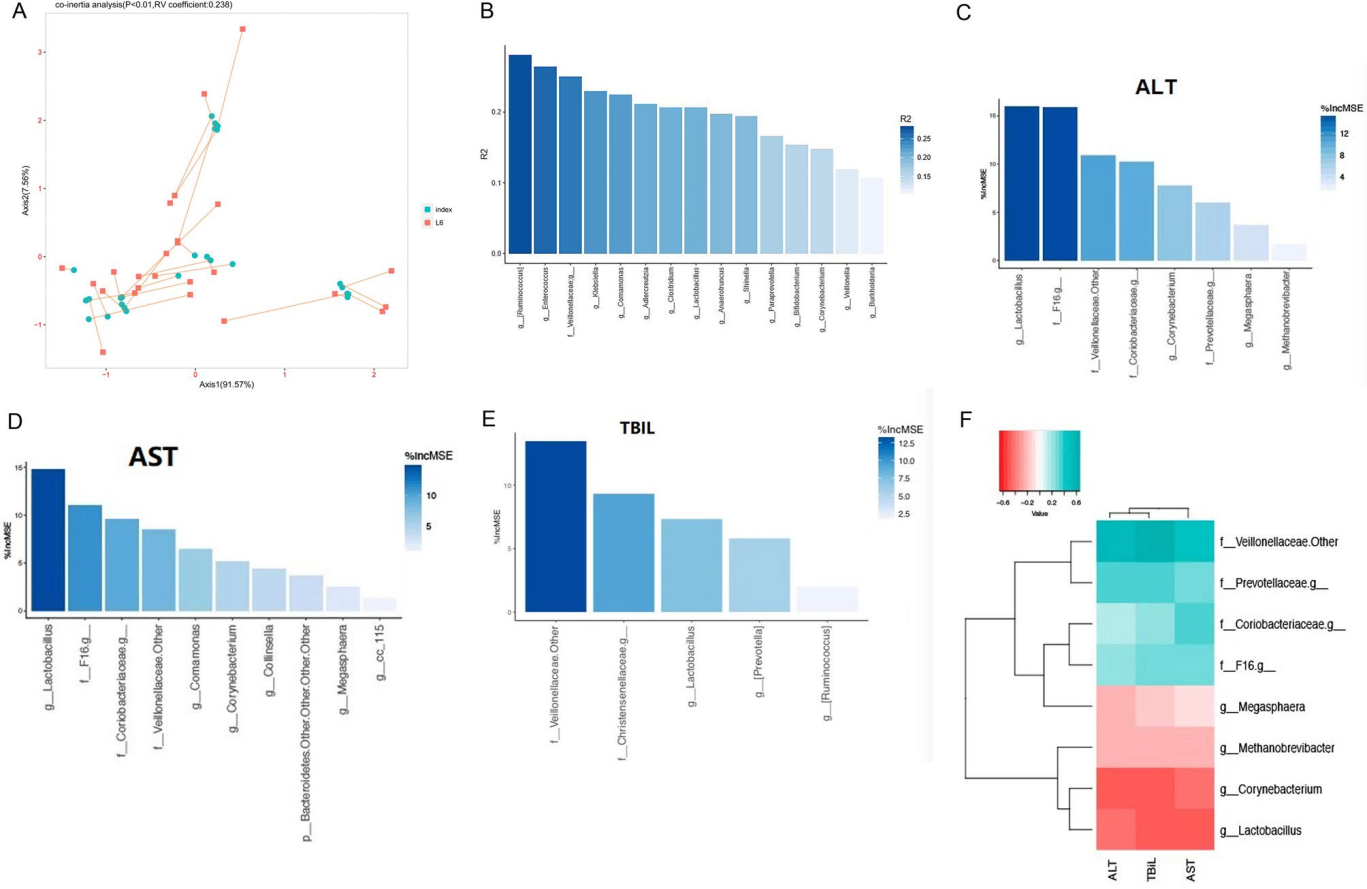
Covariant relationship of bacteria with clinical indicators. (**A**) Co-inertia analysis. (**B**–**E**) Adnois analysis. (**F**) Random forest model.

Using co-inertia (CIA) analysis, it was found that bacteria had a covariant relationship with clinical indicators. The influence of bacteria on clinical indicators was further quantified. There was a significant correlation between bacteria and phenotypes (*p* < 0.01, RV coefficient: 0.238), bacteria could explain 49.9% of changes in overall clinical indicators (Fig. 4A). Using Adonis analysis, we found a complex relationship between the microbiota and clinical indicators. Bacteria from 15 genera such as *Ruminococcus*, *Enterococcus, Veillonellaceae, Klebsiella, Comamonas, Adlercreutzia, Clostridium, Lactobacillus* was found to affect the clinical indicators. Further analysis of the first four bacteria in each group of mice showed that compared with the CTRL group, *Ruminococcus* and *Enterococcus* in the d-GalN group were significantly lower (*p* < 0.05), compared with the d-GalN group. The difference of d-GalN + SB group was statistically significant (*p* < 0.05). There was no significant difference between *Veillonellaceae* and *Klebsiella* (*p* > 0.05) (Fig. 4B–E). Through Random Forest association analysis, bacteria with obvious positive correlation with liver function were found to be *Veillonellaceae, Prevotellaceae, Coriobacteriaceae*, and *F16*, whereas those with obvious negative correlation were *Lactobacillus, Corynebacterium, Methanobrevibacter*, and *Megasphaera* (Fig. 4F).

### Fecal transplantation alleviates ALF via Th17/Treg

**Figure 5:**
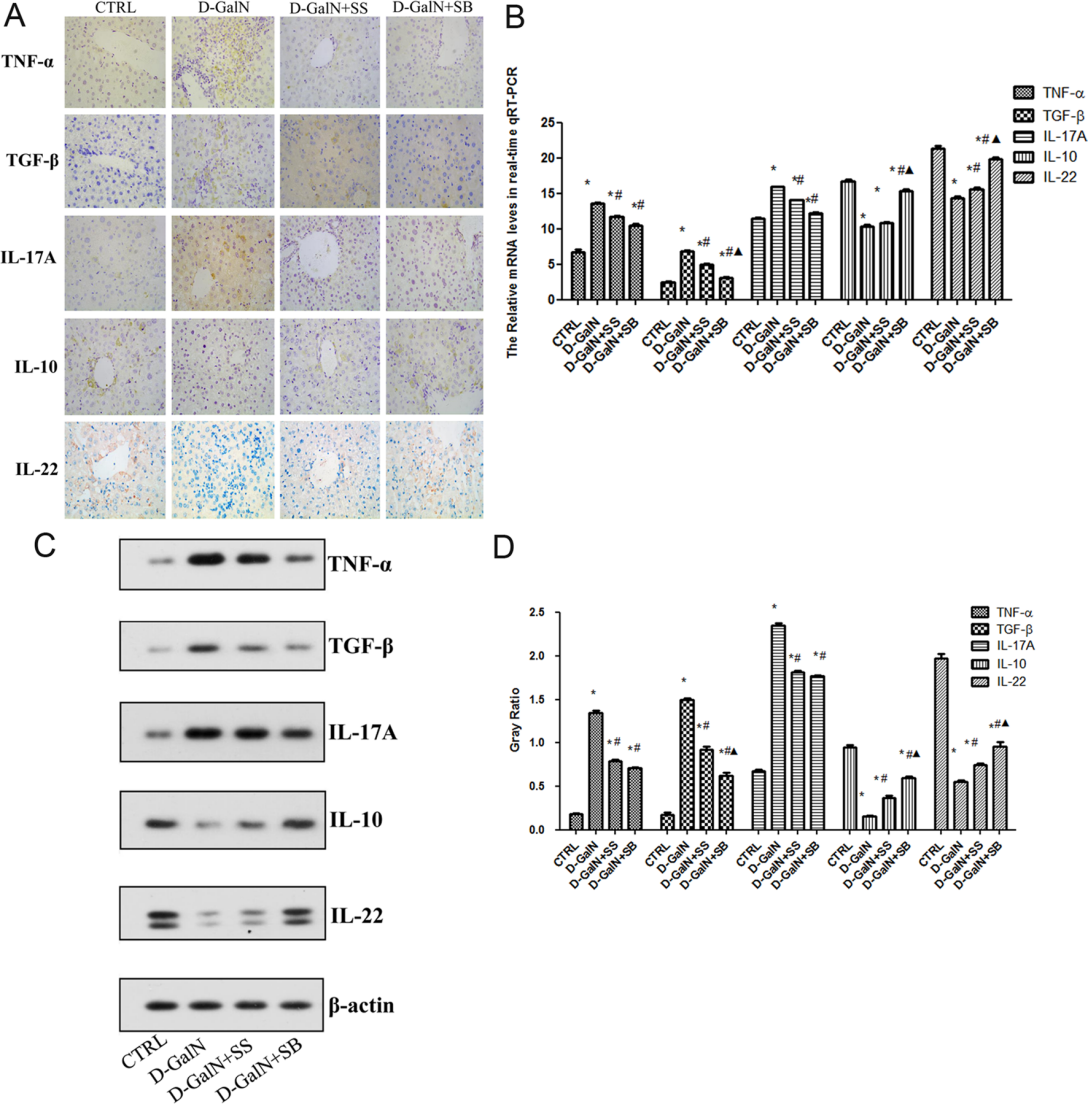
Expression levels of TNF-α, TGF-β, IL-17A, IL-10, and IL-22 in liver tissues of each group. (**A**) Immunohistochemical analysis of TNF-α, TGF-β, IL-17A, IL-10, and IL-22 in each group (× 400). (**B**) The mRNA expression of *TNF-α*, *TGF-β*, *IL-17A*, *IL-10*, and *IL-22* in each group by qPCR. (**C**) Western blotting for TNF-α, TGF-β, IL-17A, IL-10, and IL-22. (**D**) Gray ratio by western blotting. *P < 0.05 compared with control group; ^#^*P* < 0.05 compared with d-GalN group; ^△^*P* < 0.05 compared with D-GalN + SS group.

Immunohistochemical results showed that the expression of TNF-α, TGF-β, and IL-17A in the d-GalN group was higher than that in the CTRL group (Fig. 5A). The expressions of IL-10 and IL-22 in d-GalN + SS group and d-GalN + SB group were lower than that in d-GalN group. The expressions of TNF-α, TGF-β and IL-17A in the d-GalN + SB group were lower than that in the d-GalN + SS group, while the expressions of IL-10 and IL-22 were higher than that in the d-GalN + SS group (Fig. 5B–D). In addition, qRT-PCR and western blot results showed that the mRNA and protein expressions of TNF-α, TGF-β, and IL-17A in the d-GalN group were higher than those in the CTRL group (*p* < 0.05).The levels of mRNA and protein expressions of IL-10 and IL-22 in d-GalN + SS group and d-GalN + SB group were lower than those in d-GalN group (*p* < 0.05). In comparison, the mRNA and protein expressions of TNF-α, TGF-β, and IL-17A in the d-GalN + SB group were lower than those in the d-GalN + SS group, and the mRNA and protein expressions of IL-10 and IL-22 were higher than those in d-GalN + SS group, including TGF-β, IL-10, and IL-22 (*p* < 0.05, Fig. 5B–D).

## Discussion

ALF is a life-threatening condition with high morbidity and mortality rates. However, some patients have been found to spontaneously recover, suggesting that other factors other than the hepatic physiology affect the progression of ALF. The involvement of microbiota in liver diseases was largely described in rodent models or in humans^19–21^. In addition, variability in the susceptibility to develop ALF has also been observed in animal models bearing different microbiota^22^.

Given the limitations of existing ALF treatments, large efforts are now aimed at developing novel therapies. FMT is rapidly accepted as a viable, safe, and effective treatment for various liver conditions^23,24^. However, whether FMT has a healing effect on ALF has not been sufficiently investigated. In a previous study, we reported that *Saccharomyces boulardii* administration could change the gut microbiota in mice and alleviate acute liver failure^18^. The results of our current study further demonstrate that the protective function of *Saccharomyces boulardii* can be exerted not only by direct administration, but also by indirect FMT. In our animal model, d-GalN-induced abnormal aminotransferase activities and serious dissolution of the hepatocyte architecture were effectively attenuated by FMT. In addition, serum IL-10, IL-17A, and TNF-αwere significantly reduced and were found to increase after FMT intervention. Previously studies have reported increased level of TGF-β after FMT^25,26^, while we found that TGF-β was significantly reduced. It is possible that FMT is inducing an anti-inflammatory environment, thus leading to less requirement of TGF-β^27^.

To elucidate the underlying mechanisms, we examined the changes to the composition and diversity of the gut microbiota. Results showed that the proportion of certain genera in the gut microbiota was significantly changed by FMT, some of which are supported by published research findings. For example, we observed an increase in *Parasutterella* genus after FMT, which is consistent with the reported low *Parasutterella* levels in animal models of alcoholic liver disease^28^ and fatty liver disease^29^. The decrease in *Anaeroplasma* after FMT in our model is consistent with reported enrichment of *Anaeroplasma* in a nonalcoholic steatohepatitis mouse model^30^. Through CIA analysis, a significant correlation was observed between the micromicrobiota and clinical indicators, among which 49.9% clinical changes could be explained by bacteria. Further, effect-size analyses and Adonis analysis shows that 15 bacterial genera are dominant, and the most influential are *Ruminococcus* and *Enterococcus. Ruminococcus* can participate in the biometabolism, and transport and catabolism of the secondary metabolites^31^. *Enterococcus* is an intestinal bacterium found under normal physiological conditions. It ferments carbohydrates, produces lactic acid, but does not produce gas, and also ferments lactose. Analysis of *Ruminococcus* and *Enterococcus* showed that their proportion in d-GalN + SS and d-GalN + SB groups increased after FMT, and the difference in d-GalN + SB group is statistical significant (*p* = 0.012), indicating a protective effect of FMT.

In addition, we have confirmed that FMT reduced liver injury by increasing the *Lachnospiraceae* population and reducing *S24-7* population to restore intestinal microbiota composition. *Lachnospiraceae* is a probiotic that can produce short-chain fatty acids (SCFAs). Studies have shown that SCFAs can regulate Treg cells to reduce inflammation^32^. Therefore, we used qRT-PCR, western blotting, and immunohistochemistry to investigate the changes in the expression of Th17/Treg cytokines in the liver induced by fecal bacteria transplantation. IL-17A, TNF-α, and TGF-β expression in the d-GalN group was significantly higher than that in the CTRL group (*p* < 0.05), whereas IL-10 expression was significantly lower than that in the CTRL group (*p* < 0.05). Compared with that in d-GalN group, IL-17A, TNF-α, and TGF-β expression in d-GalN + BB group was significantly decreased (*p* < 0.05), whereas IL-10 expression showed a significant increase (*p* < 0.05). The results are similar to those reported by Zhou et al.^33^. Recently, a prospective randomized trial reported fecal microbiome transplant in patients with cirrhosis reduced *E. feacalis* in stool and associated with a decrease in systemic inflammation^34^. These results suggest that FMT can adjust the balance of Th17/Treg cytokines in the liver tissue to improve d-GalN induced acute liver damage.

Some limitations of this study should be addressed. The impact of FMT cannot be well separated from the impact of antibiotic pretreatment. Detailed mechanisms of FMT on ALF treatment remain a matter of speculation, thus the effects of FMT on intestinal barrier and cells need to further elucidated. Ideally we would like investigate down to a specific bacterial strain. However this is not possible for the present study.

Taken together, this study provided an in-depth analysis of gut microbiota modulations after FMT. Our results demonstrated that FMT from donors could alter the gut microbiota in mice and alleviate d-GalN-induced acute liver injury, indicating a potential therapeutic strategy. Further transcriptomic and clinical research is required to better understand the underlying mechanisms of the hepatic protective effects of FMT.

## Materials and methods

### Mice treatment and tissue sampling

Male BALB/c mice at 4 weeks of age were purchased from Guizhou Medical University (Guizhou, China) and then individually housed in plastic cages for 2 weeks at room temperature (23 °C) with an artificial cycle of 12-h light and 12-h dark. At the end of each experiment, the mice were sacrificed by cervical dislocation after anaesthetization. Fresh cecal content, plasma, and liver tissue were collected and stored at − 80 °C until analyses. The above procedures were approved by and performed in accordance with the guidelines of the Ethics Committee of the Hospital Affiliated to Guizhou Medical University.

### Study design of fecal transplantation

d-GalN was purchased from Sigma Aldrich Corporation (St. Louis, Missouri, USA) to establish a mouse model of acute liver injury. All the mice were randomly divided into the following 4 groups (N = 5 in each group): (1) mice that served as vehicle control (CTRL group); (2) mice that were treated with d-GalN (d-GalN group); (3) mice with d-GalN and fecal transplants from donor mice gavaged with saline solution (d-GalN + SS group); (4) mice that were treated with d-GalN and fecal transplants from donor mice gavaged with *Saccharomyces boulardii* (d-GalN + SB group). The d-GalN group, d-GalN + SS group, and d-GalN + SBSB group were intraperitoneally (i.p.) injected with 3.0 g/kg d-GalN, while the CTRL group were injected with saline solution.

The feces of donor mice were collected fresh every day and FMT was conducted under the aseptic conditions using a laminar flow hood. The feces from the two groups of donors were collected in cages, and 100 mg (approximately 5–6 fecal particles) was suspended in 1 mL sterile normal saline. The solution was vigorously blended for 10 s before centrifugation at 800×*g* for 3 min. The supernatant fluid (approximately 500–600 microns) was collected and fed to the mice over 10 times.

According to the established microbial depletion and recolonization protocol^35–38^, a combination of ciprofloxacin (0.2 g/L) and metronidazole (1 g/L) were added to the drinking water of FMT recipients for 2 days before FMT. Each recipient was given 100 mL of fecal supernatant orally 48 h after d-GalN injection, and was given FMT once every 24 h, a total of 7 times. The serum, liver and intestinal flora of the two groups were compared. Serum samples, liver tissue specimens, and gut microbiota were collected.

### Serum a minotransferase activities and enzyme-linked immunosorbent assay (ELISA)

Fasting blood was collected from each mouse and centrifuged at 1000×*g* for 5 min at room temperature. Serum sample was extracted and stored at − 20 °C until further analysis. Serum alanine aminotransferase (ALT), aspartate aminotransferase (AST), and total bilirubin (TBIL) activities were quantified with the enzymatic kinetic method by using a HORRON RD171 semi-automatic biochemistry analyzer (HORRON XLH Medical Electronics, China) according to the manufacturer’s protocol.

To evaluate the role of Treg/Th17-driven inflammation, serum cytokines from Treg/Th17 cells, including interleukin IL-10, IL-17A, TNF-α, and TGF-β, were measured by a commercially available ELISA kit (k3605) from Shanghai Hengke Biotechnology Co., Ltd. (Shanghai, China). Data was analyzed by microplate reader (DR-200B).

### Histologic examination

Histologic examination was carried out following our previous protocols^6^. Briefly, a 0.2 cm × 0.2 cm tissue specimen was taken from the right liver lobe from each mouse. All specimens were dehydrated by using graded solutions of alcohol, fixed in pH 7.4 and 10% buffered neutral formalin, and embedded in molten paraffin wax. After hematoxylin and eosin staining, the morphologic evaluation was performed under 10 × with a light microscope (SP2, Leica). Stained tissue sections were blind reviewed by two pathologists based on a double scoring system.

### DNA isolation and sequencing

Metagenomic DNA was extracted from the fcecal contents using a QIAamp-DNA stool minikit (Qiagen, Germany) according to the manufacturer’s instructions and previously published protocols^39^. Real-time qPCR was performed using TaqMan Universal Master Mix (Life Technologies, USA) to examine the quantity and the quality of the DNA extracted from the samples. According to the 16S metagenomic sequencing library preparation guide (Illumina, USA), total DNA was amplified using primers targeting the 16S V3 and V4 regions under the following conditions: 95 °C for 3 min, followed by 30 cycles at 95 °C for 30 s, 54 °C for 30 s, and 72 °C for 30 s, and a final extension at 72 °C for 5 min. Pooled V3-V4 amplicon libraries were sequenced using the Illumina MiSeq platform with a V3 reagent kit.

### Fecal microbial analysis

A 16S V3 + V4 region library was constructed by PCR amplification of the 16S rDNA using V3 − V4 specific primers. Sequence data for at least 50,000 paired-end read pairs from each sample were acquired using the Illumina HiSeq sequencing platform. The sequence data were then subjected to filtering for read quality and length, removing primer sequences, and splicing paired-end reads to obtain high quality data for OTU clustering and subsequent analysis. Cluster analysis of species abundance was performed using the QIIME1.91 software package, and phylogenetic groups were classified using RDP and Green gene databases.

### Real-time quantitative polymerase chain reaction (qPCR)

Total RNA was extracted from liver tissue and cDNA was synthesized using RNA reverse transcriptase. All primers were provided by Shanghai Sangon biological engineering co., LTD (Shanghai, China). The β-actin housekeeping gene was used as an internal reference to quantitatively determine the Ct value.

### Western blotting

Protein concentration in the liver lysates was determined using a BCA kit (Pierce, Rockford, IL). Lysates were electrophoresed on a reducing 12% SDS-PAGE, transferred to an Immobilon-P membrane (Millipore Corp, Bedford, MA), incubated with the antibodies of IL-10 (1:800, Beijing Boosen Biotechnology co., LTD, Beijing, China), IL-17A (1:1000), IL-22 (1:800), TNF-α (1:800), and TNF-β (1:800), and then developed using the enhanced chemiluminescence (ECL) system (Pharmacia Biotech, Piscataway, NJ). The expression of the β-actin protein expression was used as a loading control. Individual band densities were analyzed as follow: We quantified the signal intensity of a specific band on the developed image using Adobe Photoshop and then averaged the intensity readings from three independent experiments. The intensity of the image background was subtracted prior to pooling.

### Immunohistochemical staining

Immunohistochemical staining was performed on sections from paraffin embedded liver tissues, according to the method of Martinet et al.^40^. The IL-10, IL-17A, IL-22, TNF-α, and TGF-β anti-mouse monoclonal antibodies were diluted to 1:600. The sections were stained with DAB and re-dyed with hematoxylin. Images were captured via an Olympus BX51, the optical density was measured using a MIAS (MIAS 2000, Olympus, Tokyo, Japan), and the mean optical density for each section was calculated from five randomly visualized fields, under 400 ×.

### Statistical analysis

Data were analyzed via SPSS 19.0 (SPSS Inc., Chicago, IL, USA) and shown as mean ± standard deviation (SD). The groups were compared by one-way ANOVA, and *p* < 0.05 was considered to indicate a statistically significant difference. Weighted Fast UniFrac principal coordinate analysis (PCoA) based on OTUs was performed to provide an overview of gut microbial dynamics after fecal transplantation. Between-group beta-diversity was examined using LEfSe software^41^, in order to detect features with significantly different abundances between assigned classes. Analysis of bacterial population and clinical indicators using were performed using effect size analyses analysis, Adonis analysis, co-inertia analysis (CIA) analysis^42^.

## Supplementary Information

Supplementary Information.
