# Supplementary material for: Fecal transplantation alleviates acute liver injury in mice through regulating Treg/Th17 cytokines balance
Source: Sci Rep. 2021 Jan 15;11:1611. doi: 10.1038/s41598-021-81263-y (PMC7810881; doi:10.1038/s41598-021-81263-y)

**Fecal transplantation alleviates acute liver injury in mice through regulating Treg/Th17 cytokines balance**

**Running title:** Fecal transplantation alleviates liver injury

Yongmei Liu^1,2#^, Linda Fan^3#^, Zhuo Cheng^4^, Lei Yu^5^, Shuo Cong^3^, Yaxin Hu^6^, Lili Zhu^7^, Baofang Zhang^3^, Yiju Cheng^8^, Peiling Zhao^2^, Xueke Zhao^3*^, Mingliang Cheng^3*^

^1^ Department of Medical Examination，Guizhou Medical University, Guiyang, Guizhou, China

^2^ Clinical Laboratory Center, the Affiliated Hospital of Guizhou Medical University, Guiyang, Guizhou, China

^3^ Department of Infectious Diseases, the Affiliated Hospital of Guizhou Medical University, Guiyang, Guizhou, China

^4^ Department of Clinical Medicine, Peking University Health Science Center, Peking University, Beijing, China

^5^ Guizhou maternal and child health care center, Guiyang, Guizhou, China

^6^ Prenatal Diagnosis Center, the Affiliated Hospital of Guizhou Medical University, Guiyang, Guizhou, China

^7^ Department of blood transfusion, the Affiliated Hospital of Guizhou Medical University, Guiyang, Guizhou, China

^8^ Department of respiratory, the Affiliated Hospital of Guizhou Medical University, Guiyang, Guizhou, China

^#^ The authors contributed equally to this work.

* Correspondence:

Mingliang Cheng and Xueke Zhao

Address: Department of Infectious Diseases, the Affiliated Hospital of Guizhou Medical University, No. 28 Guiyang Street, Guiyang, Guizhou 550002, China

E-mail: [minglianggy@126.com](mailto:minglianggy@126.com); and Zhaoxueke1@163.com

Supplementary figure 1 Lefse data for D-GalN+SS versus D-GalN+SB


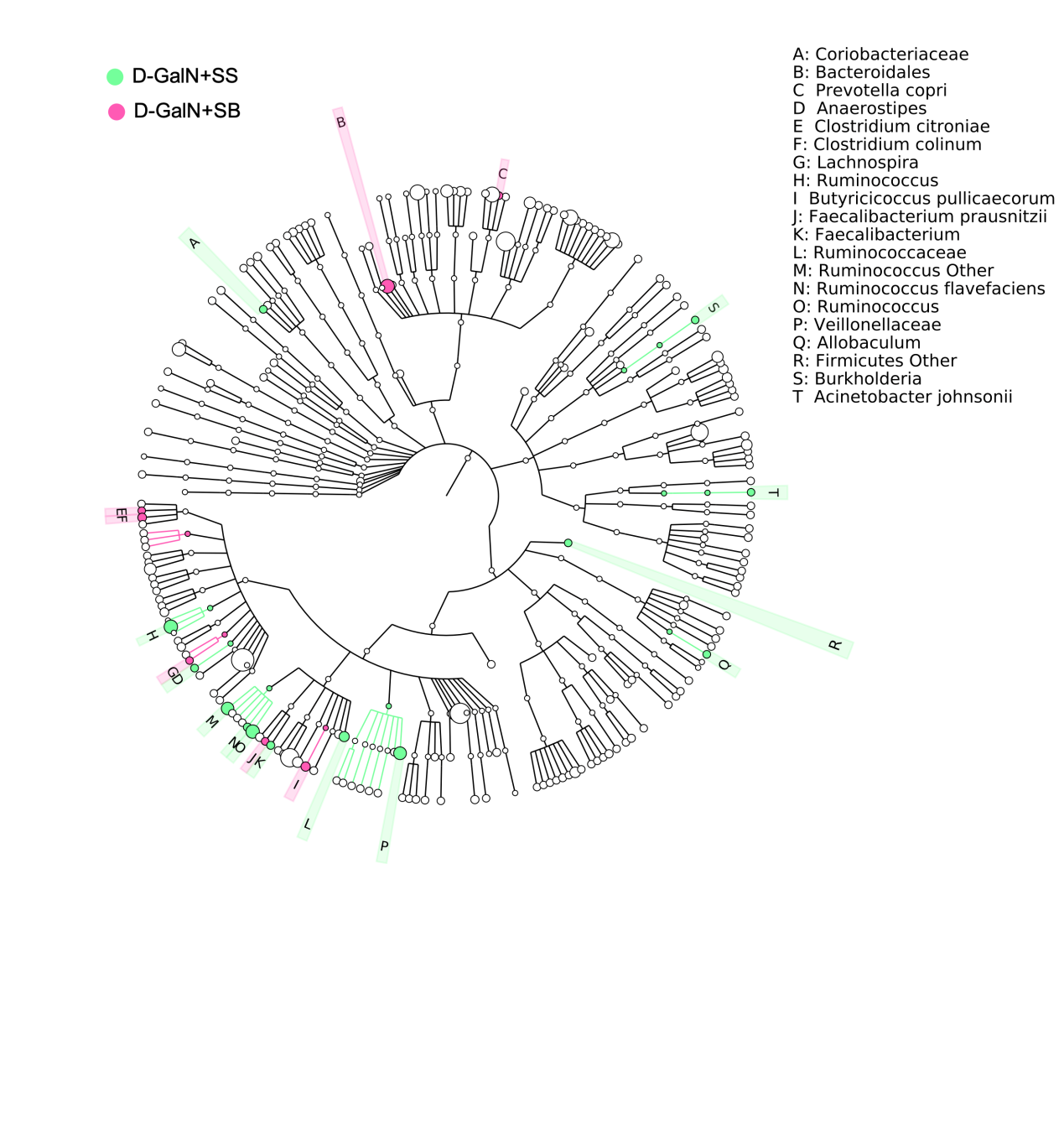


Supplementary figure 2 Lefse data for D-GalN versus D-GalN+SS


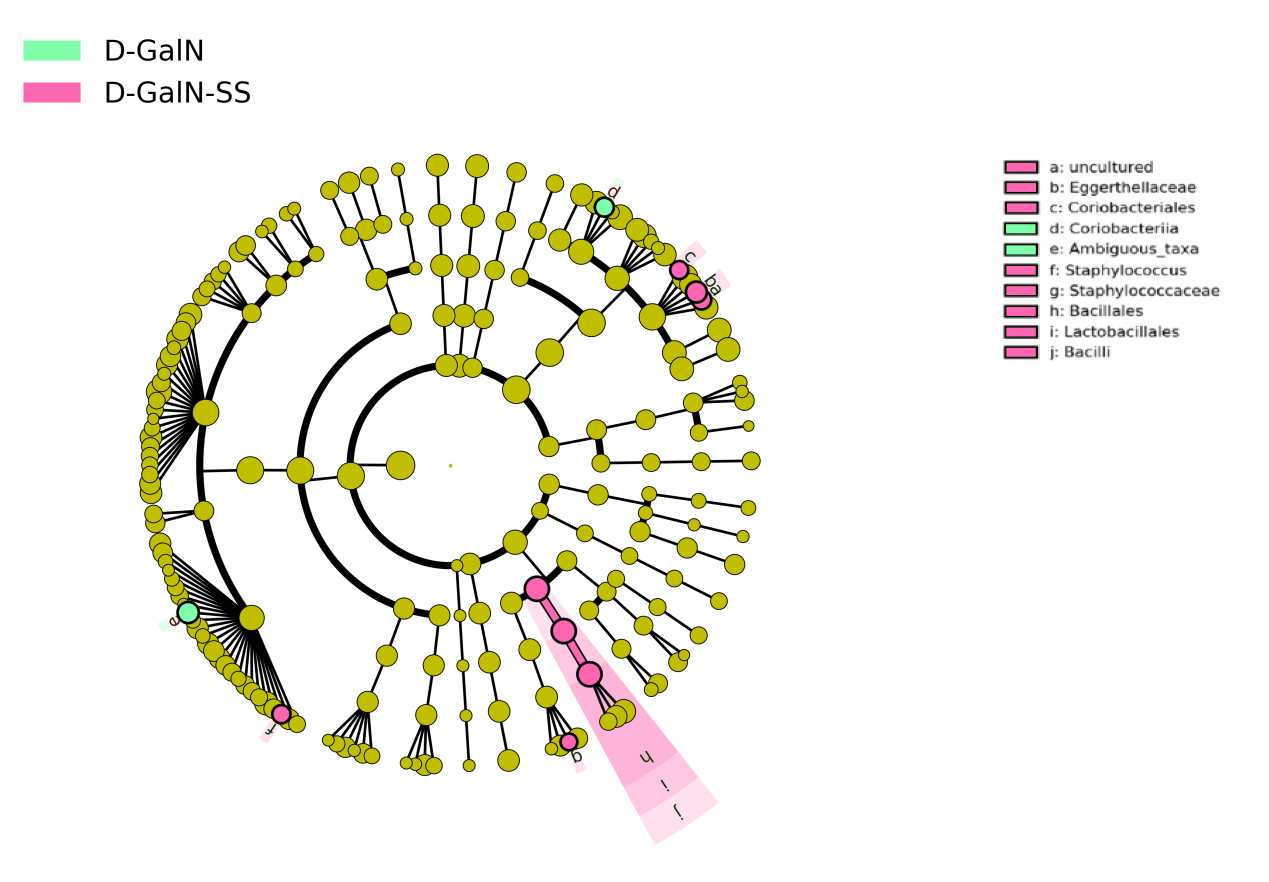


Supplementary figure 3 The uncropped images determined by western blot


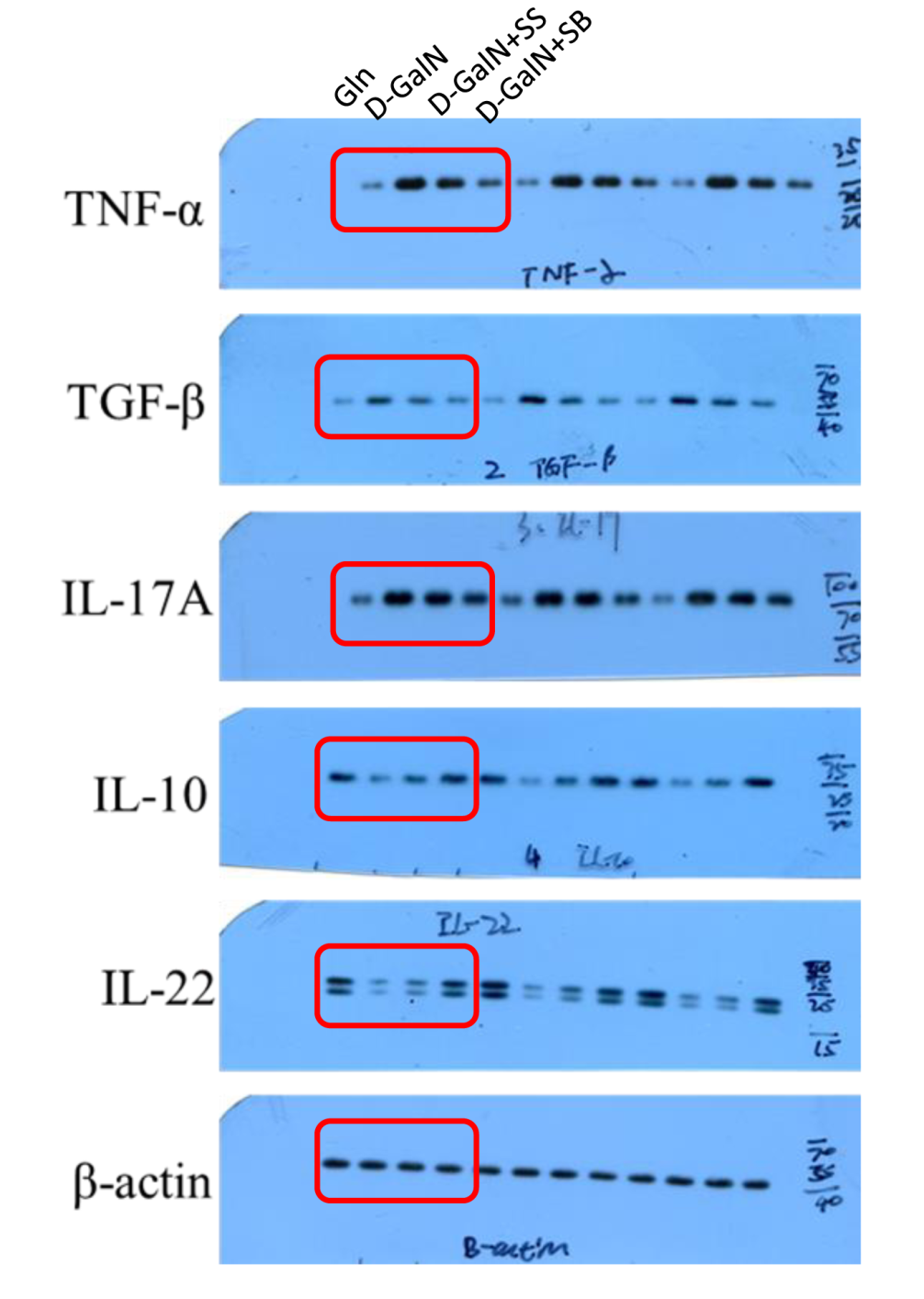

Supplement: Supplementary file 1 — Supplementary Information. [file 41598_2021_81263_MOESM1_ESM.docx]
